# Comparison of the antibody responses to *Plasmodium vivax *and *Plasmodium falciparum *antigens in residents of Mandalay, Myanmar

**DOI:** 10.1186/1475-2875-10-228

**Published:** 2011-08-06

**Authors:** Tong-Soo Kim, Hyung-Hwan Kim, Jung-Yeon Kim, Yoon Kong, Byoung-Kuk Na, Khin Lin, Sung-Ung Moon, Yeon-Joo Kim, Myoung-Hee Kwon, Youngjoo Sohn, Hyuck Kim, Hyeong-Woo Lee

**Affiliations:** 1 Department of Parasitology, College of Medicine, Inha University, Incheon 405-751, Republic of Korea; 2 Division of Malaria and Parasitic Diseases, Korea Centers for Disease Control and Prevention, Cheongwon-gun 363-951, Republic of Korea; 3 Vascular Medicine Research Unit, Brigham and Women's Hospital, Harvard Medical School, Cambridge, MA 02139, USA; 4 Department of Molecular Parasitology, Center for Molecular Medicine, Samsung Biomedical Research Institute, School of Medicine, Sungkyunkwan University, Suwon, 440-746, Republic of Korea; 5 Department of Parasitology and Institute of Health Sciences, Gyeongsang National University College of Medicine, Jinju 660-751, Republic of Korea; 6 Department of Medical Research (Upper Myanmar), Pying Oo Lwin Township, Mandalay, Myanmar; 7 Department of Gynecology, College of Oriental Medicine, Sangji University, Wonju 220-717, Republic of Korea; 8 International Research Center for Bioscience and Biotechnology, Jungwon University, Goesan 367-805, Republic of Korea; 9 Department of Pathology, University of Florida, J-566, 1600 SW Archer Road, Gainesville, FL 32610, USA

## Abstract

**Background:**

The aim of this study was to investigate the profile of antibodies against several antigens of *Plasmodium vivax *and *Plasmodium falciparum *in Mandalay, Myanmar.

**Methods:**

Malaria parasites were identified by microscopic examination. To test the antibodies against *P. vivax *and *P. falciparum *in sera, an indirect immunofluorescence antibody test (IFAT) was performed using asexual blood parasite antigens. An enzyme-linked immunosorbent assay (ELISA) was performed with circumsporozoite protein (CSP), Pvs25 and Pvs28 recombinant proteins of transmission-blocking vaccine candidates for *P. vivax*, and liver stage specific antigen-1 and -3 (PfLSA-1, PfLSA-3) for *P. falciparum*.

**Results:**

Fourteen patients among 112 were found to be infected with *P. vivax *and 26 with *P. falciparum *by thick smear examination. Twenty-three patients were found to be infected with *P. vivax*, 19 with *P. falciparum *and five with both by thin smear examination. Blood samples were divided into two groups: Group I consisted of patients who were positive for infection by microscopic examination, and Group II consisted of those who showed symptoms, but were negative in microscopic examination. In *P. falciparum*, IgG against the blood stage antigen in Group I (80.8%) was higher than in Group II (70.0%). In *P. vivax*, IgG against the blood stage antigen in Group I (53.8%) was higher than in Group II (41.7%). However, the positivity rate of the PvCSP VK210 subtype in Group II (40.0%) was higher than in Group I (23.1%). Similarly for the PvCSP VK247 subtype, Group II (21.7%) was higher than that for Group I (9.6%). A similar pattern was observed in the ELISA using Pvs25 and Pvs28: positive rates of Group II were higher than those for Group I. However, those differences were not shown significant in statistics.

**Conclusions:**

The positive rates for blood stage antigens of *P. falciparum *were higher in Group I than in Group II, but the positive rates for antigens of other stages (PfLSA-1 and -3) showed opposite results. Similar to *P. falciparum*, the positive rate of pre-blood stage (CSP VK210 and 247 subtype) and post-blood stage (Pvs25 and 28) antigens of *P. vivax *were higher in Group II than in Group I. Therefore, sero-diagnosis is not helpful to discriminate between malaria patients and symptomatic individuals during the epidemic season in Myanmar.

## Background

Malaria constitutes a major health problem and is strongly associated with socioeconomic ramifications in many temperate and most tropical countries. In Myanmar, malaria is ranked as the number one public health problem, and nearly 600,000 malaria patients seek medical attention at health institutions annually. Among malaria species in Myanmar, *Plasmodium falciparum *accounts for approximately 80% of infections and *Plasmodium vivax *for 17.8% of infections, whereas the remaining infections are due to *Plasmodium malariae *or mixed infections [1].

The sporozoites of malaria parasites are transmitted from the saliva of infected mosquitoes and stay for a while at the site of infection or travel to the liver and invade hepatocytes, where they develop into the exoerythrocytic stage called tissue schizont. During this stage, the parasites express liver stage-specific antigens. In *P. falciparum*, at least two of the relevant antigens, liver stage antigen-1 (PfLSA-1) and liver stage antigen-3 (PfLSA-3), have been identified and characterized [2-4]. These proteins are both surface proteins, are expressed solely in infected hepatocytes, and are thought to play a role in liver schizogony and merozoite release. Specific humoral, cellular, and cytokine immune responses to PfLSA-1 and PfLSA-3 are well documented, with identified epitopes that correlate with antibody production, proliferative T-cell responses, or cytokine induction [3-5]. Both pre-erythrocytic antigens have been considered as vaccine candidates against *P. falciparum *due to their antigenic and protective immunogenic properties [6-9]. In the present study, the levels of antibodies acquired against *P. falciparum *LSA-1 and LSA-3 in inhabitants of Myanmar were monitored to determine the prevalence of this parasite.

The surface membrane of all *Plasmodium *sporozoites is covered by an antigen, the circumsporozoite protein (CSP). CSP has a central immunodominant region, consisting of tandem repeats of short amino acid sequences, which contain multiple copies of the immunodominant B cell epitope [10]. Because CSP is highly immunogenic and can induce a protective response in sporozoite-immunized experimental animals and in humans, it is being investigated as a candidate for a human malaria vaccine. These immunodominant B cell epitopes of a large number of *P. falciparum *isolates of diverse geographical origin and a smaller number of *P. vivax *isolates were examined and were found to be conserved among species [11]. Two groups were identified: the dominant VK210 subtype and variant form VK247 subtype. A strain of *P. vivax *containing a variant repeat in its CSP was first isolated in Thailand [12]. The repeat of this variant strain (Thai VK247) differs at 6-9 amino acids within the repeat sequence found in all previously described *P. vivax *CSP. Following this discovery, several studies were conducted to evaluate the global distribution of the VK247 variant; it was detected in indigenous populations of China [13], Brazil [14], Mexico [15,16], Peru [16,17], and Papua New Guinea [15]. Evaluating the proportion of CSP subtypes in Myanmar will be helpful to design future vaccine applications based on CSP.

Pvs25 and Pvs28 from *P. vivax*, which were cloned from the Sal I strain, have four evolutionarily conserved tandem epidermal growth factor (EGF)-like domains attached to the parasite surface by a glycosylphosphatidylinositol (GPI) anchor. Antibodies against these proteins have the ability to block parasite formation in infected mosquitoes. These proteins have been investigated as transmission-blocking vaccines to induce an immune response in the human host that inhibits the formation of ookinetes or oocysts in malaria vectors and consequently preventing the transmission of parasites from mosquitoes to humans [18].

Additionally, an indirect fluorescent antibody test (IFAT) was used to analyse the antibodies in blood samples because serological data can provide additional evidence as to the extent and degree of malaria endemicity and reflect the period of the infection [19]. Serological surveys have provided valuable epidemiological information, especially in areas with low endemicity [20]. The rate of parasitaemia is the classical method for measuring the endemicity of malaria, whereas the incidence of parasitaemia alone can completely fail to provide an adequate description of a malaria situation in a population. When the incidence of malaria is low, mass blood surveys do not yield results commensurate with the work involved [21]. Therefore, the application of IFAT could reflect the situation in the population [22].

## Methods

### Study areas and blood sample collection

**Figure 1:**
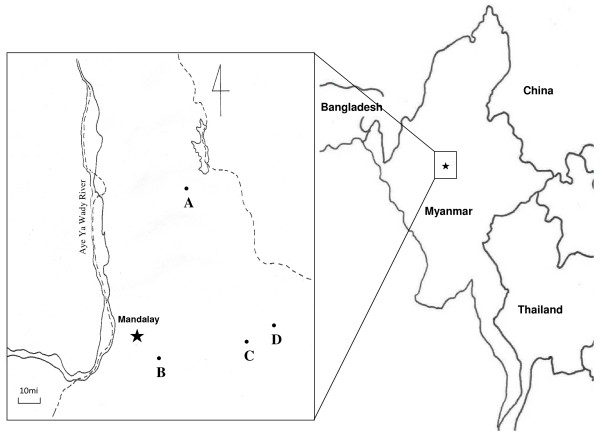
**Study areas in Mandalay, Myanmar**. A: Sedaw Gyi; B: Kyauk Me; C: Pyin Oo Lwin; D: Wet Won.

The study was conducted in Sedaw Gyi, Kyauk Me, Pyin Oo Lwin, and Wet Won in Mandalay, Myanmar, in June 2004. Sedaw Gyi is located 25 miles to the north (Figure 1A), Kauk Me is located 15 miles to the east (Figure 1B), Pyin Oo Lwin (Figure 1C) is located 42 miles to the east, and Wet Won is located 50 miles to the east of Mandalay (Figure 1D). All patients exhibited clinical symptoms associated with malaria. Thin and thick blood smears were prepared from the blood collected from the fingertips of patients for microscopic examination (magnification 7 × 100). A local health unit employee read the Giemsa-stained blood smears, and treatment was administered to those patients who tested positive for malaria, based on the guidelines of The Department of Health, the Union of Myanmar. Before treatment, additional blood samples of approximately 3 ml were collected from each patients whose infection was confirmed by microscopic examination (n = 52) or patients who had fever only (n = 60). The blood samples were transferred to the National Institute of Health, Korea Centers for Disease Control and Prevention, Repubic of Korea, for further antibody analysis. Informed consent was obtained from all patients. The study protocol was approved by the Department of Health (Upper Myanmar), the Union of Myanmar.

### Indirect fluorescent antibody test

To test for antibodies against malaria, an indirect fluorescent antibody test (IFAT) was performed with whole blood of patients infected with *P. vivax *or *P. falciparum *as described by Sulzer *et al *[23]. Briefly, 10 ml of malaria parasite-infected blood was collected from patients by vein puncture. After removing the plasma, the cells were suspended in phosphate buffered saline (PBS, pH 7.2) and centrifuged for 5 min at 2,500 rpm. The supernatant was discarded, the cells were suspended in fresh PBS, and the wash step was repeated three more times. Finally, an appropriate amount of PBS was added to maintain the parasitaemia at no less than 1%. The cells were dropped on each well of Teflon-coated slides which were dried at room temperature for 12 hrs and maintained at -70°C until required. To determine the antibody titres against *P. vivax *or *P. falciparum *for each patient, the antigen slides were fixed in pre-cooled acetone (-20°C) for 10 min and washed with PBS, and 20 ㎕ diluted sera from 1:32 to 1:8,192 (vol/vol) was added to each well. The positive and negative controls were dropped on each slide and incubated in a moisture chamber for 30 min at 37°C. The reactions were stopped by washing out the reacted sera with PBS. The slides were immersed in PBS for 6 min and then dried at room temperature. Diluted FITC conjugated anti-human IgG or IgM (Sigma, 1:32 vol/vol in PBS) was added to each well and incubated and washed using the same method described above. Several drops of buffered glycerol were then added to the samples and covered with coverslips. The slides were examined under a 40× fluorescence objective.

### Enzyme-linked immunosorbent assay

To verify that the blood samples had antibodies against the CSP VK210 [24] and VK247 subtypes [25], the Pvs25 [26] and Pvs28 [27] antigens of *P. vivax*, and the PfLSA-1 [28] and PfLSA-3 [29] antigens of *P. falciparum *(developed by the authors), an enzyme-linked immunosorbent assays were performed with these antigens. Briefly, 50 μl of capture antigen solution (0.5 μg/ml) was placed in a 96-well plate (Corning, Lowell, MA, USA) and incubated for 12 hrs at room temperature. The cells were aspirated and filled with blocking buffer (1% BSA, 0.05% PBS-Tween 20) and incubated for 1 hr at room temperature. After washing the wells three times with 0.05% PBS-Tween 20, the human serum samples in blocking buffer at a dilution of 1:100 (vol/vol) were added to each of the wells. The four positive and four negative control serum samples were also added to each plate. After a 2-hr incubation at room temperature, the plates were washed three times with 0.05% PBS-Tween 20 and then the peroxidase-conjugated anti-human IgG (Sigma, 1:2,000, vol/vol) diluted in blocking buffer was added to each well and incubated again for 1 hr at room temperature. The reaction was stopped by washing the plates as described above. To develop the colour, 100 μl 2.2'-azino-di-(3-ethyl-benzthiozoline-6-sulfonic acid) (ABTS) peroxidase substrate (Kirkegaard & Perry Laboratories, Gaithersburg, MD, USA) was added and incubated for 30 min. The absorbance of the mixture was measured at 405 nm, and the cut-off value was taken as the mean + 2 standard deviations of the negative samples.

### Data analysis

The overall proportions of infections diagnosed by each test method were compared by the Fisher's exact test. Data analyses were performed using GraphPad (GraphPad Software, Inc., La Jolla, CA).

## Results

### Detection of parasites in blood films

Three-millilitre blood samples were taken from patients who had a fever after making thick and thin smears in the field. Thick smears were immediately examined by microscopic examination. The sera were separated from blood samples and kept frozen for antibody tests. Thin blood smears were examined in more detail in the laboratory to confirm the species of parasite. As a result of the thick smear examination in the field, 14 (12.5%) patients (n = 112) were found to be infected with *P. vivax *and 26 (23.2%) with *P. falciparum*, and 72 were negative for infection (Table 1). By the thin smear technique, 23 patients were found to be infected with *P. vivax*, 19 with *P. falciparum*, five with both species, and 65 patients were negative for infection. Based on microscopic examination, blood samples were divided into two groups: Group I had a positive in thick or thin blood smear (n = 52), and Group II was negative for infection in both examinations (n = 60).

**Table 1:** Detection of malaria parasites by microscopic examination using thick and thin smears

Type of Examination	Pv (Single)	Pf (Single)	Pv + Pf (Double)^3 ^	Negative	Total
Thick^1^	14	26	0	72	112

Thin^2^	23	19	5	65	112

Pv: *Plasmodium vivax*

Pf: *Plasmodium falciparum*

^1^Thick blood smear examination

^2^Thin blood smear examination

^3^Patients who are infected with both *P. vivax *and *P. falciparum*

### Seropositive rates related to *P. falciparum *infection

When patient sera were analysed by ELISA using the PfLSA-1 and PfLSA-3 recombinant proteins as antigens, the positive rates of PfLSA-1 were similar in Group I (73.1%, 38/52) and Group II (73.3%, 44/60). The positive rates of PfLSA-3 were not significantly different in Group I (26.9%, 14/52) and Group II (31.7%, 19/60) (Table 2, *P *= 0.6444).

**Table 2:** Positive rates of liver stage-specific antigens of *P. falciparum*

	Antigen	No. Examined	No. Positive	Positive rate (%)
Group I	^1^PfLSA-1	52	38	73.1
	
	^2^PfLSA-3	52	14	26.9

Group II	PfLSA-1	60	44	73.3
	
	PfLSA-3	60	19	31.7

^1^Liver stage-specific antigen-1 of *P. falciparum*

^2^Liver stage-specific antigen-3 of *P. falciparum*

* Significant difference between Group I and Group II results (Fisher's two-tailed *P *= 0.6444).

When patient sera were analysed by IFAT using *P. falciparum-*infected red blood cells as antigens, the positive rates of IgG were not significantly different in Group I (80.8%, 42/52) and Group II (70.0%, 42/60). The positive rates of IgM were similar in Group I (21.2%, 11/52) and Group II (23.3%, 14/60) in Group II (Table 3, *P *= 0.4986).

**Table 3:** Positive rates of blood stage-antigens of *P. falciparum*

	Antibody	No. Examined	No. Positive	Positive rate (%)
Group I	IgG	52	42	80.8
	
	IgM	52	11	21.2

Group II	IgG	60	42	70.0
	
	IgM	60	14	23.3

* Significant difference between Group I and Group II results (Fisher's two-tailed *P *= 0.4986).

### Seropositive rates related to *P. vivax *infection

When patient sera were analysed by ELISA using the VK210 and VK247 recombinant proteins of *P. vivax *as antigens, the positive rates of VK210 were not significantly different in Group I (23.1%, 12/52) and in Group II (40.0%, 24/60). The positive rates of VK247 also were not significantly different in Group I (9.6%, 5/52) and Group II (21.7%, 13/60) (Table 4, *P *= 0.6551).

**Table 4:** Positive rates of Circumsporozoite protein (CSP) of *P. vivax*

	Antigen	No. Examined	No. Positive	Positive rate (%)
Group I	^1^VK210	52	12	23.1
	
	^2^VK247	52	5	9.6

Group II	VK210	60	24	40.0
	
	VK247	60	13	21.7

^1^Isolates that reacted with synthetic peptide carrying the amino acid sequence [GDRA(D/G)GQPA] of the dominant form of *P. vivax *CSP.

^2^Isolates that reacted with synthetic peptide carrying the amino acid sequence [ANGA(G/D)(N/D)QPG] of the variant form of *P. vivax *CSP.

* Significant difference between Group I and Group II results (Fisher's two-tailed *P *= 0.6551)

When patient sera were analysed by IFAT using *P. vivax-*infected red blood cells as antigens, the positive rates of IgG were not significantly different in Group I (53.8%, 28/52) and in Group II (41.7%, 25/60). The positive rates of IgM were similar in Group I (28.8%, 15/52) and in Group II (25.0%, 15/60) (Table 5, *P *= 0.8661).

**Table 5:** Positive rates of blood stage antigens of *P. vivax *by IFAT

	Antibody	No. Examined	No. Positive	Positive rate (%)
Group I	IgG	52	28	53.8
	
	IgM	52	15	28.8

Group II	IgG	60	25	41.7
	
	IgM	60	15	25.0

* Significant difference between Group I and Group II results (Fisher's two-tailed *P *= 0.8661).

When patient sera were analysed by ELISA using Pvs25 and Pvs28 recombinant proteins as antigens, the positive rates of Pvs25 were not significantly different in Group I (19.2%, 10/52) and in Group II (36.7%, 22/60). The positive rates of Pvs28 were not significantly different in Group I (23.1%, 12/52) and Group II (50.0%, 30/60) (Table 6, *P *= 0.8503).

**Table 6:** Positive rates of Transmission Blocking Vaccine candidates of *P. vivax *

	Antigen	No. Examined	No. Positive	Positive rate (%)
Group I	Pvs25	52	10	19.2
	
	Pvs28	52	12	23.1

Group II	Pvs25	60	22	36.7
	
	Pvs28	60	30	50.0

* Significant difference between Group I and Group II results (Fisher's two-tailed *P *= 0.8503).

## Discussion

Although Myanmar is one of the major malaria endemic countries and contributes to approximately 60% of death due to malaria in Southeast Asia [1], the antibody dynamics against the malaria parasites prevalent in this county are poorly understood. This study demonstrated the diversity of several antibodies against *P. vivax *and *P. falciparum *to provide an insight into the dynamics of malaria transmission, which in turn increases our understanding of vaccine application and malaria control in Myanmar.

More patients were found to be positive for infection (n = 47) by thin blood smears than by thick blood smears (n = 40) by microscopic examination; these results might be explained by spending three times more time in laboratory conditions. Additionally, it was much easier to discriminate between *P. vivax *and *P. falciparum *species by thin smear examination. Therefore, five cases of infection with both species were detected by thin blood smear examination (Table 1). Blood was taken from patients who had malaria symptoms, and the blood samples were divided into two groups based on microscopic examination; that is, positive cases (n = 52) as Group I and negative cases (n = 60) as Group II. The positive rates as measured by the antibody test with PfLSA-1 were similar in both groups. Furthermore, the positive rates of PfLSA-3 was higher in non-patient Group II than patient Group I (Table 2). However, the positive rates of IFAT (IgG detection) were higher by more than 10% in Group I (Table 3). Sero-immunological diagnosis, in particular by IFAT, is an important tool for the detection of malaria, especially when microscopic evidence of the parasites is not available due to several reasons [22]. Among those cases that were positive for *P. falciparum *infection by microscopic examination, four were negative for IgG and IgM as detected by IFAT (7.6%). These patients may not have been exposed to *P. falciparum*.

During CSP serotyping of *P. vivax*, seven individuals were positive for the VK210 subtype alone, five were positive for both the VK210 and VK247 subtypes, and no one was positive for just the VK247 subtype in Group I. Twelve patients were positive for the VK210 subtype alone, twelve were positive for both the VK210 and VK247 subtypes, and only one was positive for the VK247 subtype alone in Group II. In conclusion, the positive rates of the VK210 subtype, dormant form (32.1%, 36/112) was twice that of the VK247 subtype, variant form (18.1%, 18/112) in Groups I and II combined (Table 4). Similar to the IFAT results of *P. falciparum*, the positive rates of IgG of *P. vivax *in Group I was a little bit higher than that of Group II (Table 5).

Among the patients positive for *P. vivax *infection by microscopic examination, eight patients were negative for IgG and IgM as detected by IFAT (15.4%). These patients may have been recently infected by *P. vivax*. Notably, the positive rates of transmission-blocking vaccine candidates in Group II were double those of Group I (Table 6). The significance of this observation should be elucidated in the future studies.

## Conclusions

A major finding of our study was that the profile of antibodies against several malaria antigens, especially current vaccine candidates, in Myanmar is extremely complex. These data could be used as fundamental information for sero-epidemiological studies in Myanmar. The geographic location of Myanmar appears to contribute to the large diversity in serology of *P. vivax *and *P. falciparum *in this country. Only the blood stage antigens showed high positivity rates in Group I (patient group) for both *P. vivax *and *P. falciparum*. For other antigens, PfLSA-1 and PfLSA-3 for *P. falciparum *and VK210, VK247, Pvs25, and Pvs28 for *P. vivax*, Group II (non patient group) had higher positivity rates than Group I. Therefore, antibody detection does not in any way help to support the results of microscopic examination. A remaining unsolved question in this study is how the high positivity rate in the non-patient group prevents the onset of disease. Further studies using more blood samples are required to define the relationship between antibody production and illness.

## Competing interests

The authors declare that they have no competing interests.

## Authors' contributions

TSK, HHK, HK, and HWL conceived and designed the study and contributed to execution of research. HWL wrote the manuscript. HWL, YK, KL, BKN, YJK, and SUM collected the blood samples in the field. YS, JYK, KMC, and HK examined the thin blood smears and KL the thick blood smears. YS, YK, and HK helped in the design and implementation of field studies. TSK, HHK, JYK, MHK, and HWL carried out ELISA and IFAT. All authors have read and approved the final manuscript.
